# Circumferential Negative Pressure Wound Therapy with Instillation and Dwell Prior to Delayed Flap Coverage for a Type IIIB Open Tibia Fracture

**DOI:** 10.7759/cureus.4511

**Published:** 2019-04-20

**Authors:** Ian G Hasegawa, Patrick C Murray

**Affiliations:** 1 Orthopedic Surgery, John A. Burns School of Medicine, University of Hawaii, Honolulu, USA; 2 Orthopedics, University of Hawaii, Honolulu, USA

**Keywords:** tibia fracture, open fracture, 3b, gustilo anderson, free flap, infection

## Abstract

Gustilo and Anderson type IIIB open tibia fractures are associated with high rates of surgical site infection, wound complications, and flap failure. Controversy surrounds the optimal timing and method of wound management prior to flap coverage. No studies to date have investigated the use of negative pressure wound therapy with instillation and dwell for open type IIIB tibia fractures. We present a single case of an open type IIIB tibia fracture that was managed with 21 days of circumferentially applied negative pressure wound therapy with instillation and dwell prior to flap coverage. Our results suggest that negative pressure wound therapy with instillation and dwell may minimize infection risk, decrease wound size, and allow for delayed soft tissue coverage.

## Introduction

A systematic approach is required to avoid the high rates of wound infection and subsequent flap failure that have been reported following Gustilo and Anderson open type IIIB tibia fractures [1,2]. Typically this consists of early antibiotic administration, thorough wound debridement, postoperative antibiotics, and timely flap coverage. Often, multiple wound debridements are required prior to flap placement. Appropriate wound coverage during this transitory period is critical to reduce the risk of hospital contamination and subsequent wound infection [3].

Temporary management of type IIIB open tibial fractures with negative pressure wound therapy (NPWT) has shown some success in the past [2,4]. More recently, NPWT combined with saline or antiseptic instillation and dwell (NPWT-id) has emerged as a treatment method for acute and chronic wounds [5,6]. In a recent review, NPWT-id was shown to be superior to standard wound care therapy, including NPWT alone [5]. Surprisingly, no studies to date have reported on the use of NPWT-id for type IIIB open tibia fractures. We present a case report of an open type IIIB tibial shaft fracture that was managed with NPWT-id for 21 days prior to free flap coverage.

## Case presentation

History

A 27-year-old Caucasian male presented to the emergency department with an open right tibia and fibula shaft fractures following a high-speed motorcycle crash. The patient was helmeted at the time of the crash and there was no reported loss of consciousness. He reported isolated right lower extremity pain without neurologic complaints.

Exam and diagnostics

This was an isolated injury with no clinical or radiographic evidence of intracranial, -thoracic, -abdominal, or -pelvic injury. A FAST exam (Focused Assessment with Sonography in Trauma) was performed prior to our orthopaedic exam, which demonstrated no signs of hemorrhage.

**Figure 1:**
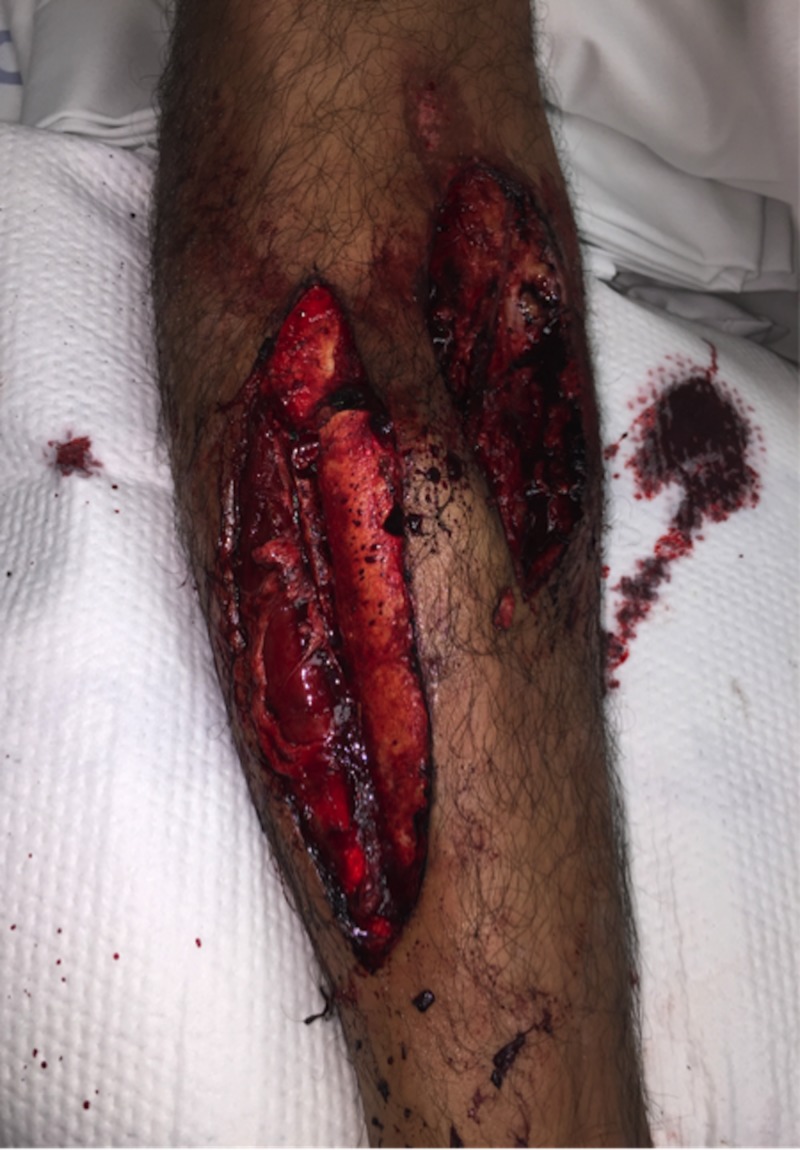
Injury photograph Right lower extremity wounds over the anterolateral and anteromedial tibia with exposed bone and gross contamination.

Inspection of the right lower extremity revealed two large wounds to the anterolateral and anteromedial tibial diaphysis. The anterolateral and anteromedial wounds measured approximately 20 cm and 12 cm in length, respectively. Both wounds exhibited gross contamination with road debris as well as exposed muscle and fracture fragments (Figure 1). There was no clinical evidence of compartment syndrome. There were no sensory or motor deficits involving the superficial peroneal, deep peroneal, or tibial nerves. A strong dorsalis pedis pulse was palpable, however the posterior tibial pulse was unidentifiable on palpation or Doppler ultrasound. A computed tomography (CT) angiogram was obtained which demonstrated vascular stenosis of the posterior tibial artery at the level of the fracture. All hematologic and metabolic labs were within normal ranges.

Time from the emergency department to the initial operative encounter

**Figure 2:**
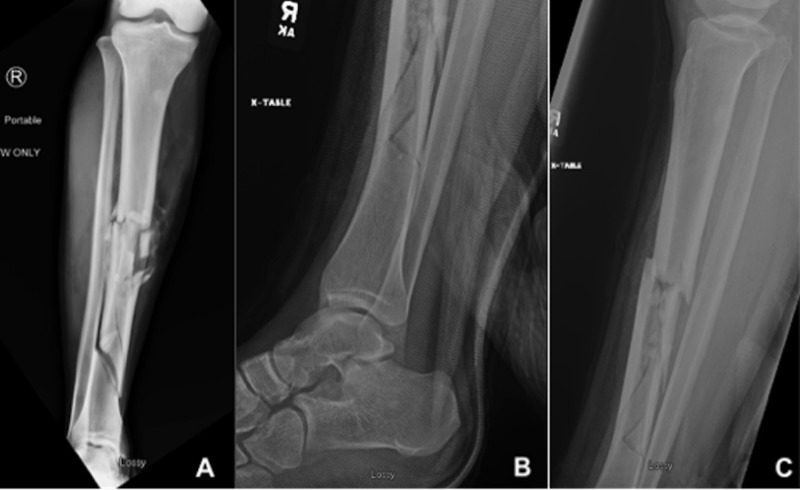
Injury radiographs (A) Anteroposterior radiograph of the right tibia and fibula, (B) lateral radiograph of the right ankle, (C) lateral radiograph of the right tibia and fibula.

Dual antibiotic prophylaxis, consisting of cefazolin and gentamycin, was administered promptly upon arrival to the emergency department. Antibiotic administration was estimated to be within three hours from the time of injury. A brief bedside irrigation with 3 L of sterile saline was performed and the wounds were dressed with moist gauze. The patient was then provisionally stabilized with a moldable long leg fiberglass splint and sent for additional preoperative imaging. Preoperative radiographs are provided in Figure 2A-2C. After imaging was completed, the patient was brought to the operating room for urgent wound debridement and open reduction internal fixation of the right lower extremity.

Initial operative encounter

**Figure 3:**
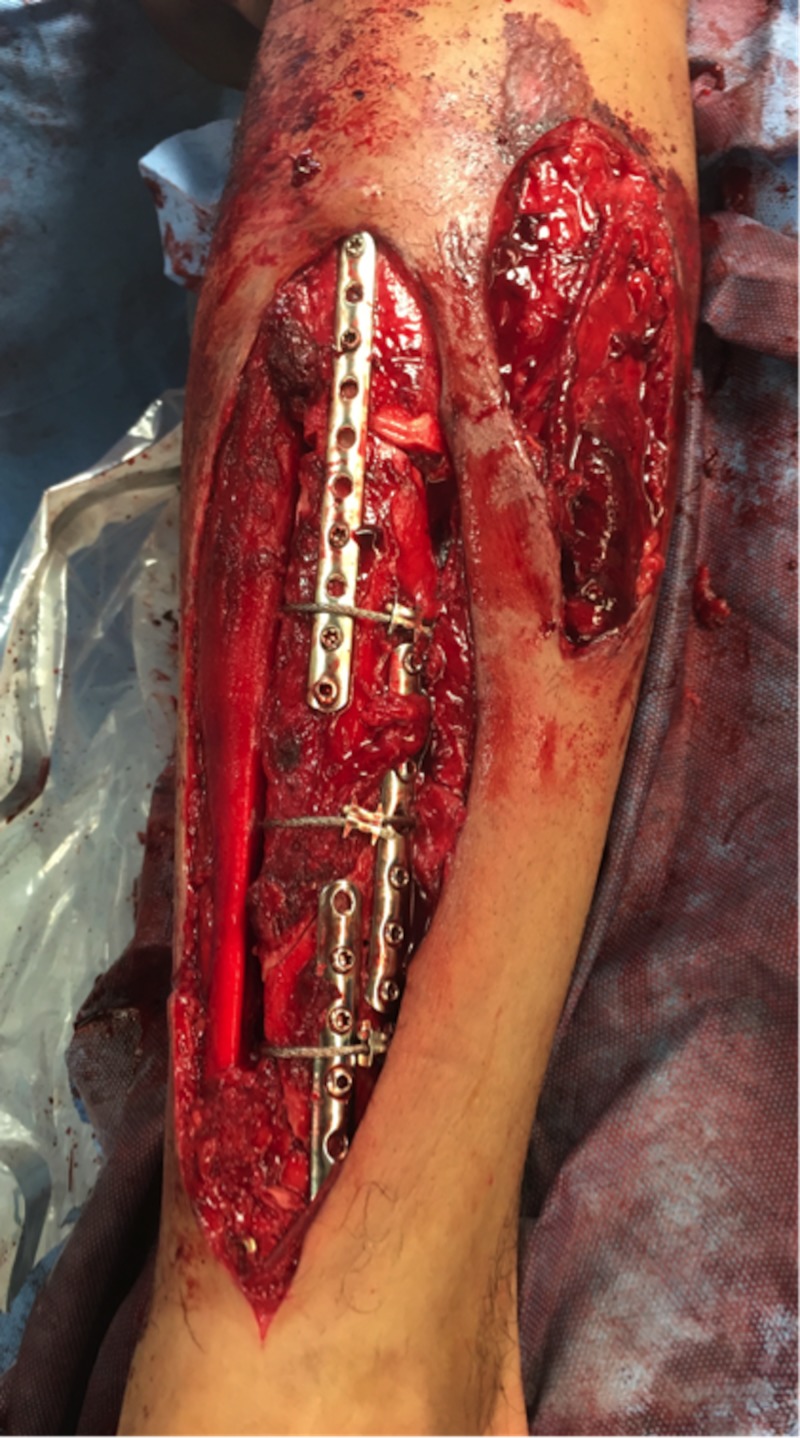
Initial wound debridement and fracture stabilization Intraoperative photograph of the right lower extremity wounds following initial wound debridement and fracture stabilization. The skin bridge separating the anterolateral and anteromedial wounds was later excised during the second wound debridement.

**Figure 4:**
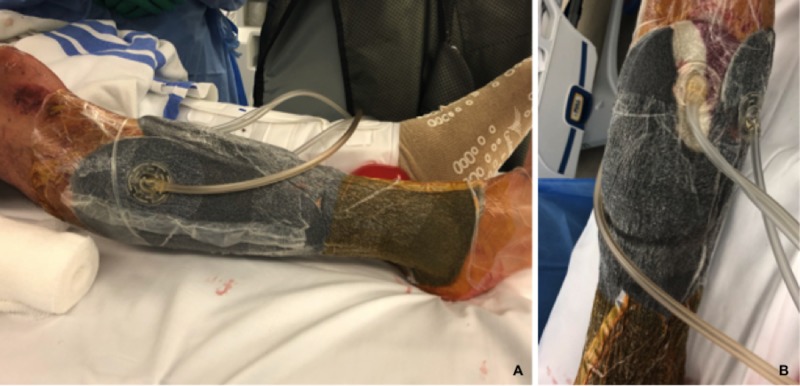
Circumferential application of negative pressure wound therapy with instillation and dwell (A) Lateral view, (B) medial view.

**Figure 5:**
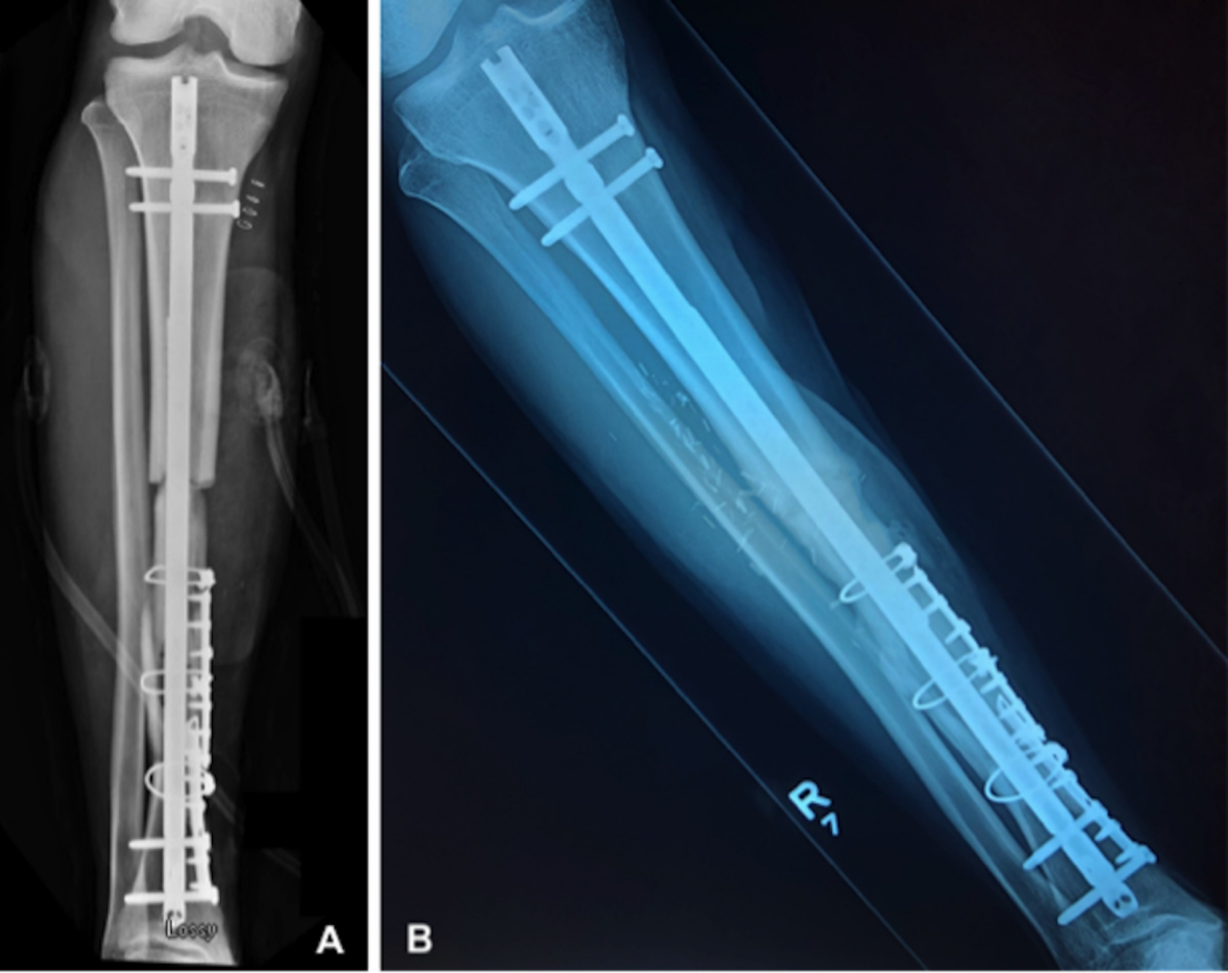
Postoperative radiographs (A) Immediate postoperative anteroposterior radiograph, (B) anteroposterior radiograph at one year follow-up.

The patient was placed under general anesthesia. A thigh high tourniquet was placed prior to the sterile preparation and draping of the surgical field. Gross wound contaminants were removed sharply including all devitalized bone. The superficial and deep peroneal nerves were identified and found to be grossly uninjured. Nine liters of sterile saline was then used to irrigate the wound. Next, the segmental tibia fracture was provisionally stabilized with multiple one-third tubular plates and unicortical 3.5 mm screws. A reamed intramedullary tibia nail was then introduced via a suprapatellar approach. Once in place, multiple cerclage wires were applied for further stabilization of large fracture fragments (Figure 3). An additional 3 L of sterile saline was used to irrigate the wounds. Once complete, all wounds were left open and a circumferential VeraFlo (Acelity, San Antonio, TX, USA) wound vac was applied (Figure 4A-4B). See technique described below. Postoperative radiographs are provided in Figure 5A.

Circumferential wound vac technique

The anterolateral and medial wounds were lined with one inch adhesive draping strips. The wounds were then lightly packed with Cleans Choice sponge (Acelity, San Antonio, TX, USA) and sealed with adhesive drapes. Cleans choice sponge was placed directly over exposed bone and hardware. When sealing the sponge, care was taken to lay drapes over the skin with minimal skin tension. Once sealed, instillation was set at a volume of 50 mL, at 10-minute soak times, cycled every three hours. Suction was set at -125 mmHg. Instillation consisted of Prontosan Irrigation Solution (B. Braun Medical Inc., Bethlehem, PA, USA) for three days followed by sterile saline for an additional four days. This allowed for a total of seven days of NPWT-id between wound vac exchanges. Next, one-inch adhesive strips were used to line the proximal and distal tibia circumferentially. Negative pressure sponge dressing was then applied circumferentially within this region and sealed with adhesive drapes. Again, care was taken to create as little tension on the skin when sealing the wound vac sponge. Once sealed, suction was set at continuous -125 mmHg.

Hospital course

Postoperatively the patient received 72 hours of intravenous cefazolin. Prophylactic anticoagulation was started on postoperative day one and continued until 24 hours prior to each subsequent return to the operating room. The patient performed daily physical and occupational therapy. The right lower extremity was restricted to partial weight bearing. However, no range of motion restrictions was implemented.

**Figure 6:**
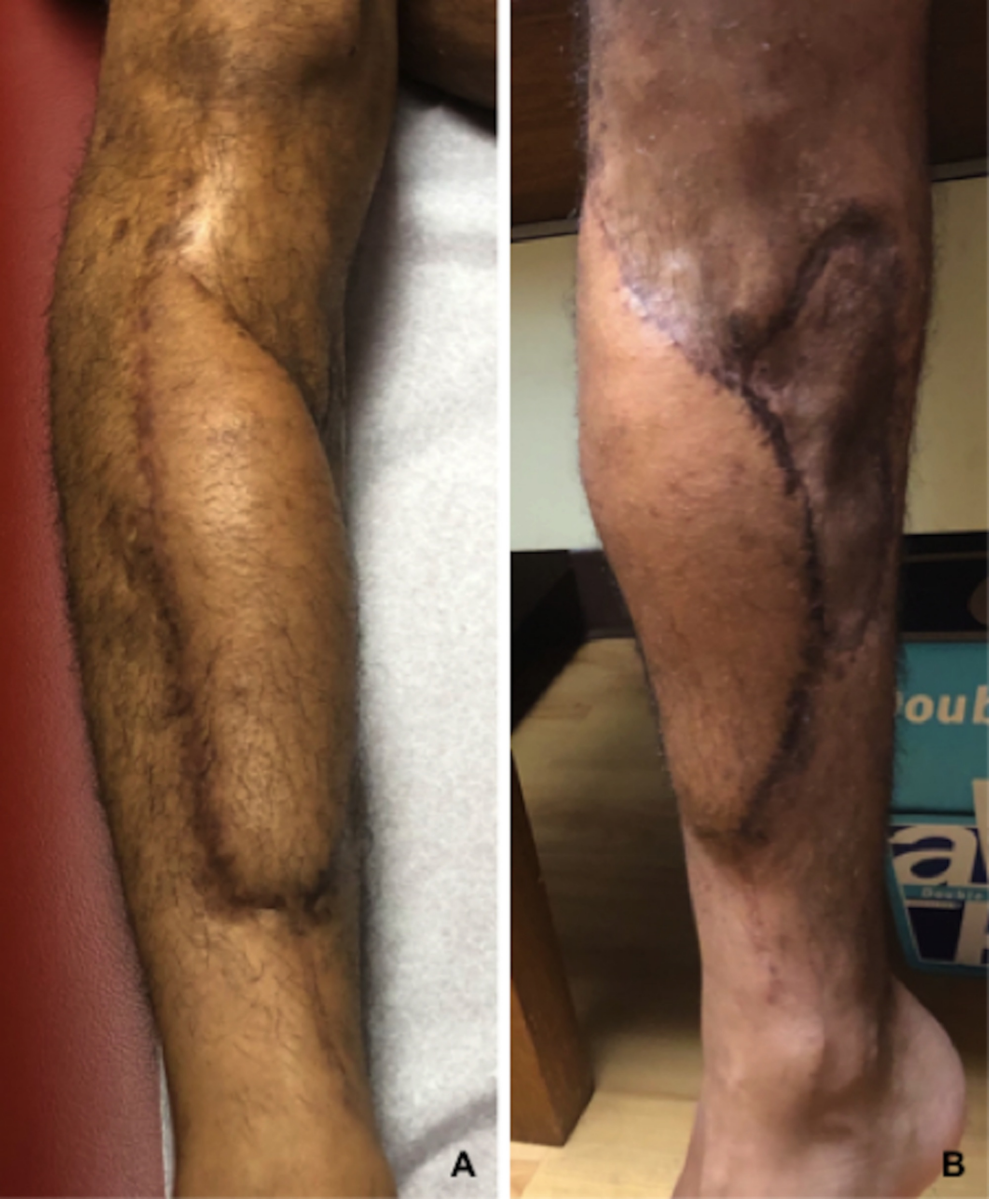
Anterolateral thigh free flap at one-year follow-up (A) Anterior view, (B) medial view.

The patient returned to the operating room two additional times for a total of three wound debridements and two wound vac exchanges prior to flap placement. Each wound vac exchange consisted of the same circumferential application and instillation and dwell settings as previously described. Time between each return to the operating room was on average seven days (i.e., hospital day one, eight, fifteen). During the first repeat wound debridement (hospital day eight), we excised a necrotic skin bridge separating the anterolateral and medial wounds. The single wound consequently measured roughly 20 cm x 20 cm at its longest and widest points. Assessment of the wound each week demonstrated progressive wound bed granulation and a decrease in overall wound size. More importantly at no time point was there concern for superficial or deep wound infection. This included physical (e.g., erythema, malodor, gross purulence) and hematologic (e.g., rising white blood cell count, sedimentation rate, C-reactive protein) signs. At three weeks from the initial injury (i.e., hospital day 22), the patient returned to the operating room for placement of an anterolateral thigh (ALT) free flap. The size of the flap measured approximately 15 cm x 10 cm. Postoperatively the patient remained in the hospital for seven days for flap monitoring. Outpatient follow-up ensued at six weeks, three and six months, and one year. At the one-year follow-up there was complete healing of the fracture site and ALT flap (Figure 5B and Figure 6A-6B).

## Discussion

To date, this is the first study to report on the wound management of an open type IIIB tibia fracture exclusively with NPWT-id prior to flap coverage. Our major findings were the absence of infection at any time point, a dramatic decrease in wound size, and an uncomplicated delay in flap coverage despite a highly contaminated wound, Tscherne grade III.

Infection and flap failure are well known complications following open type IIIB tibia fractures. As pointed out by Patzakis et al. wound contamination following open fractures likely occurs during the acute hospitalization period as opposed to the time of injury [3]. NPWT applied under sterile conditions should theoretically prevent subsequent infection by isolating the wound from hospital environmental exposure. Surprisingly, however, infection and flap failure rates following type IIIB open tibia fractures have ranged between 34-46% and 9-14%, respectively, despite management with NPWT [2,7].

The safety of prolonged NPWT has been questioned. Several studies have linked NPWT greater than seven days prior to flap coverage for type IIIB tibia fractures with higher rates of infection [7,8]. On the other hand, prolonged NPWT may be associated with a reduction in wound size and fewer flap procedures being performed [2,9,10]. We utilized a total of 21 days of NPWT prior to flap coverage without any infectious complications. This is an important finding as delayed flap coverage is an often unavoidable consequence following high-energy mechanism injuries due to time needed for resuscitation and medical optimization. We also demonstrated a dramatic decrease in wound size. We placed an ALT free flap that measured nearly half the size of the wound after the second debridement.

The optimal timing between wound vac exhanges has not been established. However, standard NPWT protocols without instillation and dwell typically implement a two to three day interval between scheduled exchanges [2,7-10]. In contrast, we implemented a weekly wound vac exchange frequency. This was done to minimize return trips to the operating room as well as to reduce the risk of hospital contamination that may occur during bedside wound vac exchanges.

It is unclear how our instillation and dwell protocol impacted our outcomes, as this was not directly tested. It is possible that our instillation and dwell protocol facilitated bacteria removal from the wound bed. Previous studies have demonstrated reduced bacterial bioburden in acute and chronic wounds following NPWT with antiseptic instillation and dwell [6,11]. Future studies would benefit from a comparison between NPWT-id and NPWT alone to assess the true efficacy of instillation and dwell.

We are also the first to report on the circumferential application of any form of vac therapy. NPWT is thought to act by improving local tissue perfusion, decreasing wound edema, and stimulating tissue granulation [12]. It is possible that our circumferential application may have enhanced these responses, in addition to the removal of bacteria as previously mentioned, given the larger regional surface area covered.

We recognize there are limitations to our study. Primarily, our study design consisting of a single case report limits the generalizability of this study. It is also questionable whether our instillation and dwell protocol consisting of three days of an antiseptic solution followed by four days of saline is more or less effective than either alone. As such, in addition to those already stated, future studies should include larger populations, and a comparison between saline and antiseptic solutions.

## Conclusions

This is the first case to report on the wound management of a type IIIB open tibia fracture exclusively with NPWT-id. While further studies are required to assess the true efficacy of our methods, our results suggest that circumferentially applied NPWT-id is not only safe, but may be associated with a reduced risk of infection, smaller wound size needing coverage, and allow for delayed soft tissue coverage.
